# Telomerase Inhibition in the Treatment of Leukemia: A Comprehensive Review

**DOI:** 10.3390/antiox13040427

**Published:** 2024-03-30

**Authors:** Elżbieta Bartoszewska, Klaudia Molik, Marta Woźniak, Anna Choromańska

**Affiliations:** 1 Faculty of Medicine, Wroclaw Medical University, Mikulicza-Radeckiego 5, 50-345 Wroclaw, Poland; elzbieta.bartoszewska@student.umw.edu.pl (E.B.); klaudia.molik@student.umw.edu.pl (K.M.); 2 Department of Clinical and Experimental Pathology, Division of General and Experimental Pathology, Wroclaw Medical University, Marcinkowskiego 1, 50-368 Wroclaw, Poland; marta.wozniak@umw.edu.pl; 3 Department of Molecular and Cellular Biology, Wroclaw Medical University, Borowska 211A, 50-556 Wroclaw, Poland

**Keywords:** leukemia, natural substances, telomerase, antioxidant, hematological malignancy, genomic instability, DNA damage, polyphenols, vitamins, in vitro, in vivo, clinical trials

## Abstract

Leukemia, characterized by the uncontrolled proliferation and differentiation blockage of myeloid or lymphoid precursor cells, presents significant therapeutic challenges despite current treatment modalities like chemotherapy and stem cell transplantation. Pursuing novel therapeutic strategies that selectively target leukemic cells is critical for improving patient outcomes. Natural products offer a promising avenue for developing effective chemotherapy and preventive measures against leukemia, providing a rich source of biologically active compounds. Telomerase, a key enzyme involved in chromosome stabilization and mainly active in cancer cells, presents an attractive target for intervention. In this review article, we focus on the anti-leukemic potential of natural substances, emphasizing vitamins (such as A, D, and E) and polyphenols (including curcumin and indole-3-carbinol), which, in combination with telomerase inhibition, demonstrate reduced cytotoxicity compared to conventional chemotherapies. We discuss the role of human telomerase reverse transcriptase (hTERT), particularly its mRNA expression, as a potential therapeutic target, highlighting the promise of natural compounds in leukemia treatment and prevention.

## 1. Introduction

Leukemia ranks as the thirteenth most prevalent cancer globally. It is classified into four primary subtypes—acute lymphoid leukemia (ALL), chronic lymphocytic leukemia (CLL), acute myeloid leukemia (AML), and chronic myelogenous leukemia (CML)—based on the predominant cell type (lymphoid or myeloid) and the pace of disease progression (acute or chronic) [1]. Among these subtypes, ALL mainly affects children and stems from the uncontrolled proliferation of bone marrow hematopoietic precursor cells. While advancements in therapeutics have improved overall survival rates, factors like lineage switching and the emergence of mixed lineages during relapses or chemotherapy often alter the prognosis of the disease [2].

Chromosome translocations involving mixed lineage leukemia 1 (MLL1, also known as MLL or KMT2A) are a significant factor in approximately 70% of leukemia cases in infants and 5–10% in children and adults, with a generally unfavorable prognosis. For MLL1-rearranged (MLL1-r) ALL, five-year survival rates are around 35%, a stark contrast to the approximately 90% for other pediatric ALLs. Similarly, MLL1-r AML exhibits poor clinical outcomes, with five-year survival rates at approximately 30%. While strides have been made in comprehending the biology of MLL1-r leukemias, a pressing need remains for more effective treatments to improve patient outcomes [3,4].

The roles of telomeres and telomerase in driving uncontrolled cellular proliferation, immortalization, and the onset of cancer are pivotal. Telomerase, in particular, has been extensively studied in various cancer types, including leukemias, due to its involvement in carcinogenesis. The dysregulation of the human telomerase gene is a well-documented event in the development of leukemia [2]. The role of MLL gene translocations in the pathogenesis of acute leukemia is significant, often leading to a bleak prognosis. MLL frequently forms fusion proteins with transcription cofactors such as AF4 (~35%), AF9 (25%), or its paralog ENL (10%). The AF9/ENL proteins, primarily through their AHD domain, play a pivotal role in forming super elongation complexes (SEC) containing AF9/ENL/AF4/AFF4. These complexes facilitate the catalytic activity of DOT1L, which is crucial for MLL-rearranged leukemia. The last studies have shown that the activity of a novel small-molecule inhibitor in disrupting the protein–protein interactions (PPI) between AF9/ENL and DOT1L/AF4/AFF4 is promising. This disruption prevents the formation of SEC and the recruitment of DOT1L to MLL target genes, leading to reduced H3K79 methylation. This molecular intervention significantly curtails the expression of malignant genes in MLL-rearranged leukemia, impedes cell proliferation, and fosters differentiation and apoptosis. In preclinical studies, the inhibitor mentioned demonstrated potent antitumor activity in a mouse model of MLL-r leukemia without causing noticeable toxicity. Its efficacy makes it a valuable tool for in vivo biological investigations and positions it as a promising candidate for further drug development efforts [5,6].

**Figure 1:**
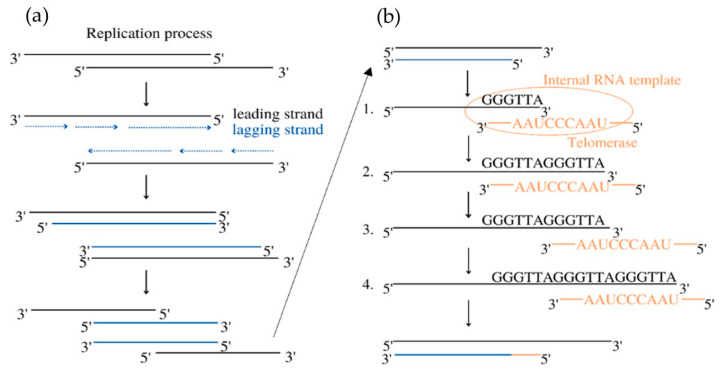
Mechanism of telomerase activity at the end of the replication cycle. Column (**a**): During the replication, the lagging strand is created based on the leading strand, which was present in the nucleus. The new strand is complementary to the old one but has its length shortened, which is the reason for cell aging. The elongation of the lagging strand is an extra step that requires telomerase and does not happen in every human cell. Column (**b**): 1. The 3′ end of the G-overhang positions on the RNA template in TER via base pair formation at the active site of TERT. 2. The addition of nucleotides creates the telomeric DNA repeat. 3. The telomerase translocates to resume telomeric repeat synthesis. 4. The elongated DNA strands are ready [10].

Telomerase is a chromosome-stabilizing enzyme, active at the end of a mitotic cycle—its main purpose is to add the missing section of the DNA on the delayed strand. It works on telomeres located at the 3′ ends of eukaryotic chromosomes, whose role is to protect the DNA against possible damage during copying, as shown in Figure 1. In somatic cells, it is impossible to completely replicate them, resulting in their shortening during every cell division [7]. Telomerase is a large complex composed of RNA and proteins. It creates multiple copies of a repetitive sequence, called the telomeric repeat, which is rich in guanine, using telomerase reverse transcriptase (called hTERT in humans) and its telomerase RNA (TER), which includes the template that coordinates the repeat synthesis (hTERC—human telomerase RNA component) [8]. These repeats are combined by the shelterin complex, composed of six proteins: protection of telomeres 1 (POT1), telomere repeat factor 1 and 2 (TRF1, TRF2), tripeptidyl peptidase I (TPP1), TRF1-interacting nuclear protein 2 (TIN2), and repressor/activator protein 1 (RAP1) [9].

In most somatic cells, telomerase activity is not found, which makes their prolific ability limited. Enzyme expression was found in germ cells and self-renewing tissues, such as the intestinal epithelium, the ovary, and hematopoietic stem cells [11]. Significant telomerase activity is expressed in approximately 90% of cancer cells and tumor tissues, which leads us to suspect the correlation between the addition of telomeric DNA and the infinite proliferation of cancer cells [12]. Further research into the mechanism underlying its regulation can be beneficial and used for better cancer diagnosis and treatment.

The extent of hTERT mRNA expression strongly corresponds to cellular telomerase activity. This indicates that this specific subunit is a key determinant of telomerase engagement in replication and may be the best option to choose while attacking this enzyme [11]. Understanding this protein allows the creation of more precise therapies that do not negatively affect the whole body. TERT function varies and may influence the process of elongation due to the creation of structural variants and genetic or epigenetic alterations. Moreover, it also affects the nucleus, cytoplasm, and mitochondrion, causing DNA damage response, stress protection, gene expression regulation, and a decrease in apoptosis [10]. TERT and TERC are accompanied by auxiliary proteins that are necessary for telomere homeostasis, which is essential in the regulation of aging and cancer formation, such as the structure-stabilizing proteins called dyskerin (DKC1) and non-histone chromosome protein 2 (NHP2). Other proteins also found are telomerase Cajal body protein 1 (TCAB1), heat shock protein 90 (HSP90), serine, and arginine-rich splicing factor 11 (SRSF11) [13].

More than 80% of tumors use diverse regulatory techniques, known as telomere maintenance mechanisms (TMMs) because they sustain telomere length by reactivating telomerase. TMMs are unique for tissue histotype, cancer type, and cell lines. The most important TTMs are *TERT* and *TERC* gene amplification, *TERT* gene rearrangements, *TERTp* somatic mutations, and transcription factor binding. Epigenetic alterations, alternative splicing, and polymorphic variants also exist within the *TERT* gene body and *TERTp*. About 10–15% of cancer cells achieve immortality utilizing a telomerase-independent mechanism, called the alternative lengthening of telomeres (ALT) [14]. The lack of telomerase activity results in the remodeling of the catalytic proteins by chromatinization or methylation [15]. The remaining percentage of tumors proliferates based on a non-defined telomere maintenance mechanism (NDTMM), in which both TERT expression and ALT are absent [10].

Telomerase inhibitors may be divided into two categories: natural or synthetic. The main substances that draw the attention of researchers are, in the case of natural inhibitors, curcumin and indole-3-carbinol, and when it comes to synthetic ones, BIBR1532 and geldanamycin. In this study, we will discuss the natural inhibitors of telomerase, which are included in Table 1 and Table 2, and show their potential as novel mono- and multitherapy ingredients.

The inhibition process varies depending on the substance—it may bind directly to the enzyme and down-regulate its activity just like oxoisoaporphine [16] or it activates intracellular pathways affecting telomerase, which happens in the instance of genistein [17]. We have remarked that each substance’s inhibitory ability is different. 

The aim of this review is to display telomerase inhibition as a promising target in cancer therapy.

## 2. Materials and Methods

**Figure 2:**
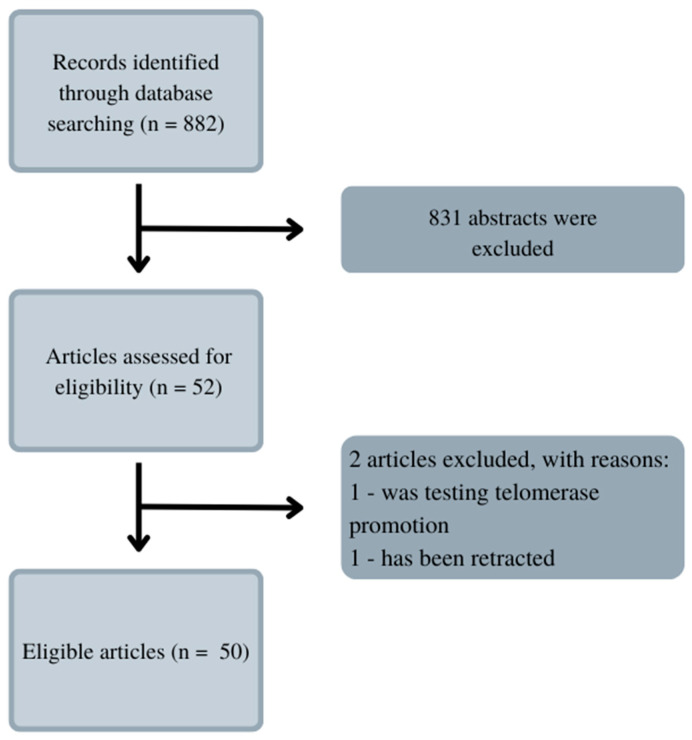
Literature research diagram. The data were narrowed down to 50 articles focusing solely on natural inhibitors.

Telomerase inhibition is an interesting prospect that has been inspiring numerous scientists to the usage of various substances. Electronic searches were conducted to find them. PubMed was the indexing database because it contained the most significant number of articles on the analyzed topic. The same analyzed articles are available in the Scopus and Web of Science databases. After searching on PubMed.gov the keywords ‘telomerase‘, ‘inhibition’, ‘leukemia’, and ‘natural substances’, there were not any articles found. The search was then changed, and the keywords were ‘leukemia’ and ‘telomerase’. This procedure allowed us to find 822 results, as shown in Figure 2. The data were narrowed down to articles focused solely on natural inhibitors. Through some articles, we found even more substances, which are mentioned in the paragraphs below. 

## 3. Natural Substances Inhibiting Telomerase

### 3.1. Polyphenols and Flavones

#### 3.1.1. Polyphenols

Polyphenols are the substances found in fruits and vegetables. Over the past number of years, researchers around the world studied their beneficial properties, currently with a special focus concerning cancers. Their anti-inflammatory, anti-carcinogenic, and antioxidant properties have been proven in numerous studies [18]. Polyphenols are the item of research as the new antibacterial therapy; they also may be useful in the prevention of neurodegenerative diseases [19,20]. Moreover, polyphenols can reduce allergenicity or act as adjuvants in sunscreen, as they have the ability to absorb UV radiation [21,22]. In our review, we wanted to draw attention to their telomerase inhibition property in leukemias.

**Figure 3:**
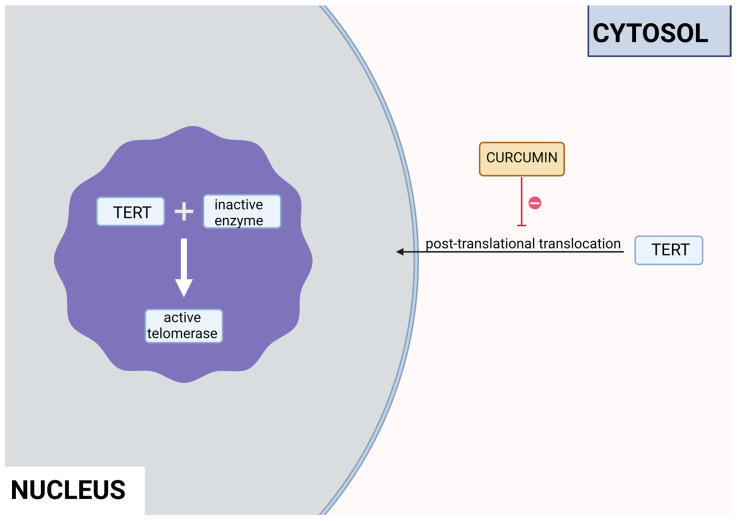
Inhibition of the post-translational translocation of the TERT cytosolic fraction in the presence of curcumin.

Curcumin is a polyphenol produced by plants from the *Curcuma longa* species and may be found in turmeric. It has many beneficial properties for the human organism such as anti-inflammatory, anti-carcinogenic, anti-mutagenic, and anti-proliferative properties. In the study performed by S. Mukherjee et al. on acute myeloblastic leukemia, the HL-60 cell line inhibition of telomerase was observed. Different concentrations of curcumin were applied to the cells that were incubated for 24 h. After this time, telomerase activity was measured using TRAP assay. It has been noticed that the inhibition of telomerase activity increases with a dose of curcumin—by 55% and 78% using 10 μM and 55 μM, respectively. The IC_50_ value was estimated at 9.8 μM. It was concluded that there is a linear relationship between the inhibition of telomerase activity and the induction of apoptosis, as curcumin also induced cytochrome c release from mitochondria to cytosol, which is an indicator of apoptosis; Bcl-2 expression decreased as well, whereas the Bax level increased [23]. Another study also indicated the induction of apoptosis by curcumin in cells from the HL-60 cell line. M. Dikmen et al. have established that apoptosis is at a significant level with 15 μM of curcumin (81.1% of late apoptotic cells) and higher concentrations are more effective—at 40 μM, the levels reach 88.6% of late apoptotic cells. Higher caspase 3 activity was also observed [24]. In the human chronic myelogenous leukemia cells from the K-562 cell line, curcumin-induced telomerase inhibition was also examined by S. Chakraborty et al [25]. Different concentrations of curcumin were applied, and telomerase activity was measured after 48 h with TRAP assay. It was found that the inhibition grows in a dose- and time-dependent manner. After 48 h, the inhibition was at 45% and 83.5% in the cells with 10 μM and 50 μM of curcumin, respectively. After 24 h with a 50mM concentration, the inhibition was at 66%. The correlation of telomerase inhibition with the proportion of apoptotic cells, DNA content in the sub-G1 peak, and caspase 3 and caspase 8 activations was shown. Furthermore, the TERT subunit was detected using Western blot. The inhibition of the post-translational translocation of the subunit from the cytosol to the nucleus was observed (the process presented in Figure 3). The expression of the cytosolic fraction of TERT was more pronounced—73.8% with the presence of curcumin, whereas without curcumin, it was at 43.2% [25].

Epigallocatechin-3-gallate (EGCG) is the main polyphenol present in green tea. In the study on the HL-60 cell line, no hTERT gene down-regulation was observed; however, apoptosis did occur due to other pathways—perhaps due to a higher Bax-Bcl2 ratio or ROS production [26]. In another study, on ALL Jurkat E6.1 cells, apoptosis was also present. The concentrations of EGCG that were used (50 μM, 70 μM, and 100 μM) induced the apoptosis of 31%, 40%, and 71% of cells, respectively [27]. However, a chemically modified derivative of EGCG (MST-312) has been found effective as a telomerase inhibitor. With the combination of MST-312 and doxorubicin, a chemotherapy medication used to treat ALL, the down-regulation of the hTERT gene was observed. Two pre-B ALL cell lines were put under investigation—NALM-6 and REH. The dosages effective toward the NALM-6 cell line were 2 μM of MST-312 and 20nM of doxorubicin, whereas toward the REH line, 4 μM of MST-312 and 5nM of doxorubicin were effective [28].

Indole-3-carbinol (I3C) is a polyphenol responsible for the indirect inhibition of telomerase activity. It may be found in cruciferous vegetables: broccoli, cauliflower, Brussels sprouts, cabbage, or kale. In the study performed on the NALM-6 cell line, 60 μM of I3C induced levels of the wild-type p53 after 24 and 48 h. p53 is a well-known telomerase inhibitor, so qRT-PCR confirmed the down-regulation of the hTERT gene. That process is crucial for the p53-dependent apoptosis in the leukemic cells [29]. In another study, the influence of I3C on the chronic myeloid leukemia cells from the K562 cell line was examined. Caspase activation triggered the apoptosis of the cells. Meanwhile, I3C caused the down-regulation of c-Myc—a transcription factor that regulates the hTERT gene transcription. That process also inhibited the activity of telomerase. The most effective concentration of I3C was 400 μM of the substance after 32 h; it has to be noted that 200 μM had no effect on c-Myc. The expression of c-Myc was measured after 18 and 32 h; however, 32 h of incubation proved to be more efficient [30].

Butein (3, 4, 2′, 4′-tetrahydroxychalcone) is a polyphenolic substance found in the stem bark of cashews and the heartwood of *Dalbergia odonifea*, *Rhus verniciflua,* and *Caragena jubata*. It is believed to exhibit anti-tumor activity. The study performed on the U937, THP-1, HL60, and K562 cell lines not only had pro-apoptotic properties but also inhibited telomerase activity. It was observed that 20 μM of butein down-regulated the *hTERT* gene by four times in the THP-1 cell line after 24 h. The process was not cell line-specific—the down-regulation of telomerase activity was caused by the suppression of the *hTERT* gene on the transcriptional level, in a p53-independent manner. In the same study, it was observed that butein inactivates Akt, an up-regulator of telomerase activity, through the phosphorylation of hTERT. The levels of p-Akt were decreased in a dose-dependent manner after 24 h [31].

#### 3.1.2. Flavones and Flavonolignans

Genistein is an isoflavone found in such plants as lupin, fava beans, soybeans, and psoralea. In the study performed on the cells from the HL-60 cell line, both the cell viability and the down-regulation of the hTERT gene were noticed. The induction of apoptosis increased by 4,25-fold, with an IC_50_ of 50 μM. The activity of the hTERT decreased in a dose- and time-dependent manner. Compared to the control group, it decreased the most: 5,16-fold at 24 h [17]. 

Apigenin (4′,5,7-trihydroxyflavone) is a flavonoid that can be found in vegetables, like onions and parsley; fruits, like oranges; herbs, like basil and oregano; and plant-based beverages, like wine and tea. It has antioxidant, inflammatory, and autoimmune properties and can be used in therapy for neurogenerative diseases and several types of cancer [32]. A study on human leukemia cells U937, HL-60, and THP-1 showed apigenin-induced toxicity. This substance activates the caspase pathway and through caspase-dependent apoptosis and suppressed *hTERT* expression, it lowers the level of telomerase activity. Moreover, it increases the intracellular level of ROS [33]. 

Silymarin is a substance extracted from milk thistle (*Silybum marianum*). It is composed of flavonolignans, like silybin. This substance acts as an antioxidant and has immunomodulatory, anti-cancerous, anti-inflammatory, and hepatoprotective effects [34]. A study on K562 cells was conducted in order to establish the anti-proliferative effect of silymarin. This substance lowers the activity of telomerase and suppresses cell growth. The lowered telomerase level was connected with silymarin-induced apoptosis [35].

Baicalin is a flavonoid substance isolated from *Scutellaria baicalensis*. It has hepatoprotective, antibacterial, and anti-inflammatory properties. In a study carried out on the HL60 cells, it was concluded that 20 μg/mL of baicalin used for different durations of time down-regulates c-Myc mRNA and protein levels. As a result, the hTERT mRNA expression was also decreased as it is a transcriptional target of c-Myc [36].

### 3.2. Vitamins

#### 3.2.1. Vitamin A

**Figure 4:**
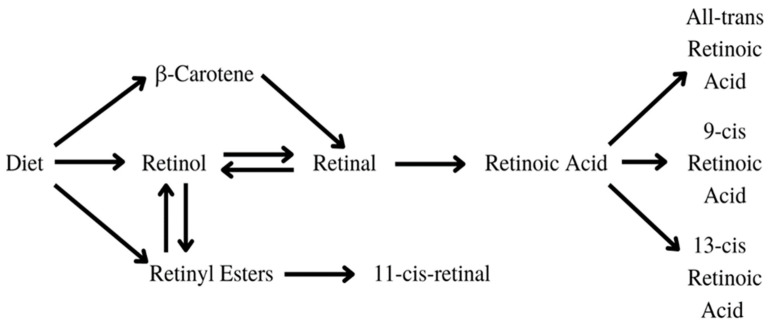
Transformation of carotenoids [5,13].

Vitamin A is a vital nutrient that serves several purposes in the human body [37]. It is crucial in embryonic development and growth, the formation of organs, immune function, vision, and reproduction [38]. The substances found in food, such as carotenoids, retinol, and retinyl esters undergo sufficient changes in the human body to be transformed into active metabolites, as displayed in Figure 4. Retinoic acid, especially all-trans retinoic acid (ATRA), exhibits anti-cancer effects by inhibiting telomerase, reducing cellular proliferation, and activating apoptosis, differentiation, and cell growth arrest [39]. 

**Figure 5:**
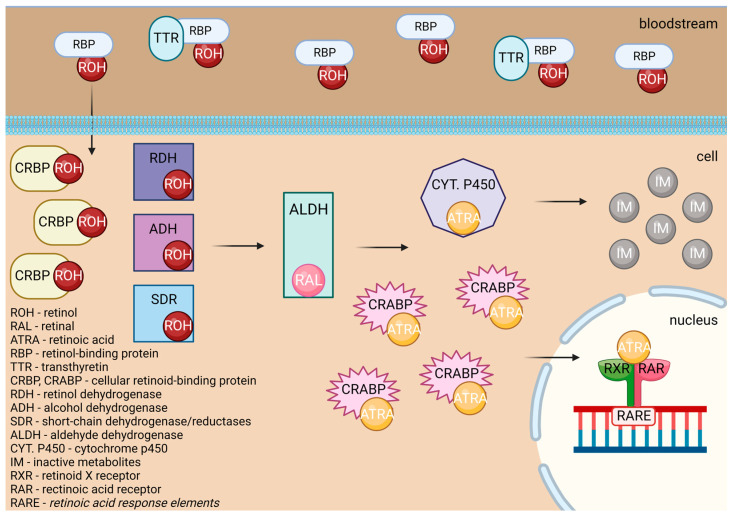
Transformation of retinol to ATRA, which leads to cellular changes.

Retinoic acids are essential in myeloid differentiation. They regulate the target gene expression through their agonistic nuclear receptors: the retinoic acid receptor (RAR), the retinoid X receptor (RXR), and their isoforms. Retinol undergoes various changes inside the human body, as shown in Figure 5. Retinol is carried through the organism bound to transthyretin (TTR) and retinol-binding protein (RBP), or RBP alone. After being carried to the cell by transthyretin (TTR) or retinol-binding protein (RBP), retinol crosses the cell membrane and bonds to cellular retinol-binding protein (CRBP). Later, it is oxidized to the retinal by retinol dehydrogenase (RDH), cytosolic alcohol dehydrogenase (ADH), or short-chain dehydrogenase/reductases (SDRs). After that, it is transformed by the aldehyde dehydrogenase (ALDH) enzyme family members into trans-retinoic acid [40].

ATRA either attaches to CRABPs and is transported into the nucleus or is destroyed by ATRA-degrading cytochrome P450 reductases such as CYP26A1, which turns it into inactive metabolites. In the first case, it acts as a ligand and binds to a heterodimer of RXR and RAR. In the regulatory region of target genes, the RAR/RXR heterodimer binds to retinoic acid response elements (RAREs), which results in a conformational change by triggering the release of corepressors and the engagement of co-activator complexes [40].

Studies using the HL-60 cell line demonstrate that ATRA’s telomerase inhibition appears quite early after its treatment, and it is involved in early differentiation. This is thanks to the abrupt reduction in *hTERT* gene transcription. A marker for the granulocytic differentiation of HL-60 cells was discovered due to the observation of the expression of integrin α-M protein (CD11b). Through that, it was found that the down-regulation of telomerase precedes the differentiation of HL-60 cells. These findings imply that ATRA’s anti-leukemic action may be mediated by its capacity to drive the differentiation of promyelocytic leukemia cells through the down-regulation of the *hTERT* gene. That gene seems to be the main target of ATRA’s control of cellular differentiation [40]. Apart from ATRA, other substances also influence retinoic acid receptors: the novel retinoids, such as 9cUAB30, induce apoptosis and inhibit telomerase by down-regulating the *hTERT* gene in HL60 cells [41]. It was also discovered that recombinant human insulin-like growth factor-binding protein 7 (rhIGFBP7) triggers the ATRA-driven eradication of leukemia stem/progenitor cells in acute myeloid leukemia (AML) with elevated *retinoic acid receptor α* gene (*RARA*) expression. Research on non-APL AML blasts shows the synergy between ATRA and rhIGFBP, which binds to growth factor independent 1 transcriptional repressor protein (GFI1), causing its down-regulation, and leading to cell death. Moreover, it increases cells’ susceptibility to ATRA, causing ATRA-induced differentiation, cell elimination, and senescence. It seems to be a prospective treatment for people with various types of AML [42].

**Figure 6:**
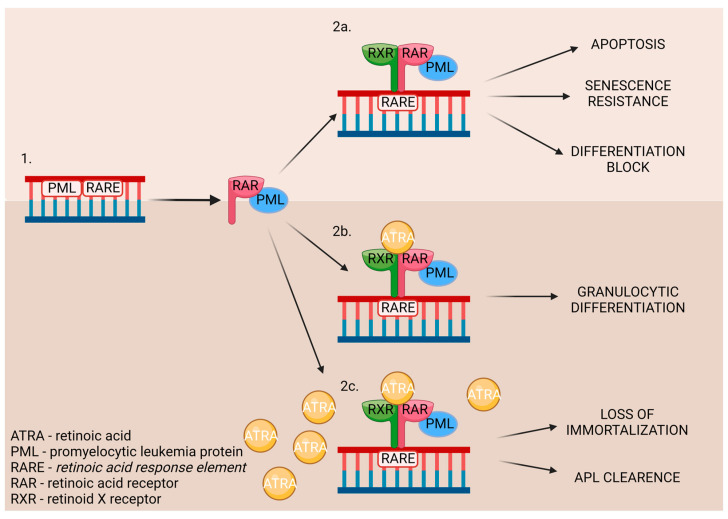
The influence of ATRA on DNA, which is used whilst treating APL patients. 1. Patient’s DNA. 2a. Treatment without ATRA. 2b. Treatment with ATRA. 2c. Treatment with a big dosage of ATRA.

Chromosomal translocation occurs in acute promyelocytic leukemia (APL), thereby causing the rearrangement of retinoic acid receptor α [41]. It involves *promyelocytic leukemia* genes and the *RARA* gene [40]. As a result, myeloid differentiation is stopped at the promyelocytic stage due to the disruption of the RA signal pathway. The pharmacological dosage of ATRA binds to the *RARA* directly and affects promyelocytic leukemia/retinoic acid receptor α (PML-RARα). It also influences communication with the nuclear receptor–corepressor complex, restoring the wild-type RARα/RXR regulatory pathway. Moreover, it promotes the downstream genes’ transcriptional expression [41]. The ATRA’s influence on the DNA is displayed in Figure 6.

When the DNA mutation occurs (1.), the promyelocytic leukemia protein (PML) nuclear bodies are created. While untreated (2a.), it leads to the development of APL. The treatment may consist of a low (2b.) or high (2c.) dose of ATRA, which triggers different outcomes in patients [43].

This mechanism shows its effectiveness in numbers—95% of patients experience complete remission with just ATRA and chemotherapy [40]. The standard treatment schedule consists of an induction period, in which the dosage is two times 45 mg/m^2^/day of ATRA and 0.15 mg/kg/day of arsenic trioxide (ATO). It is followed by four periods of consolidation, in which ATRA and ATO are also implemented. Patients with low/intermediate risk are treated with a combination of ATRA and ATO or, if ATO is unavailable, ATRA and chemotherapy. For high-risk patients, the treatment is the same, but with added gemtuzumab ozogamicin (GO) or idarubicin (IDA). Although ATO’s mechanism of action is still not fully known, studies indicate a major synergism between ATO and ATRA in cell differentiation and apoptosis in promyelocytic leukemia [44]. In pediatric APL, the standard treatment has ATRA in every stage [45] and the studies demonstrate that ATO may soon be included in the therapy as well [46]. The lack of retinoids prolongs chronic lymphocytic leukemia, and it is suggested to include a retinoic acid-rich diet in the treatment [47].

#### 3.2.2. Vitamin D

**Figure 7:**
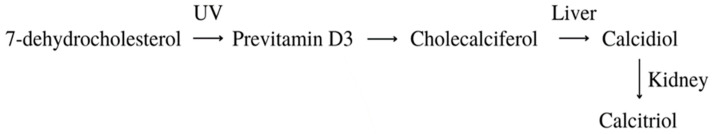
Transformation of vitamin D [49].

Calcitriol is an active form of vitamin D that can be synthesized in the human body through a series of changes, as shown in Figure 7. It presents various anti-cancer effects, such as growth inhibition, apoptosis activation, and the differentiation of several kinds of malignant cells [48]. Moreover, it sensitizes cancer cells to various treatments and lowers pro-inflammatory cytokine production [49]. Apart from its multiple anti-cancer properties, it also regulates calcium and phosphate homeostasis and participates in bone mineralization, insulin secretion, and blood pressure regulation [50]. Unfortunately, its application in therapy is limited due to its calcemic properties. This was the reason for the creation of vitamin D_3_ analogs, such as EB 1089 (1,25 dihydroxy-24,26,27-trishomo-22E,24E-diene-cholecalciferol)—they display improved antitumor activity while lowering calcemic complications. They also lower their binding affinity to the vitamin D-binding protein (DBP), which results in their easier cellular intake [48].

Vitamin D can be gained through the supplementation of specific pills, dietary intake, and exposure to ultraviolet radiation (UVR). UVR mobilizes the conversion of 7-dehydrocholesterol to pre-vitamin D_3_, which is then converted to cholecalciferol. It is carried through the bloodstream to the liver via DBP. The next form, calcifediol (25-hydroxyvitamin D), is produced thanks to cytochrome P450 2R1 (CYP2R1) hydroxylation. This form circulates in the blood and when delivered to the kidney, the cytochrome P450 27B1 (CYP27B1) transforms it to calcitriol (1,25-hydroxyvitamin D) [51]. This, now active, form affects a variety of tissues and biological pathways. Calcitriol binds the vitamin D receptor (VDR) and causes a conformational shift in the protein, which allows interaction with the RXR. The VDR-RXR heterodimer then works as a transcription factor by binding vitamin D response elements (VDRE) in DNA, which triggers target gene expression or inhibition [52]. In the case of *hTERT*, suppression is activated, which causes a lower telomerase level [53].

Cellular studies show that calcitriol regulates both normal and leukemic cell maturation. The configurations of vitamin D derivatives and retinoids display cooperative effects on differentiation in established leukemia cell lines, such as HL-60 and U937. Through ATRA-triggered pathways, vitamin D induces apoptosis in HL-60 cells and influences the expression of apoptosis-related gene products. It also impacts the generation of endogenous nitric oxide by elevating tumor necrosis factor-α protein (TNF-α) levels or by means of a secondary mediator, like the C-type lectin CD23 [54]. The novel vitamin D_3_ molecule 1,25(OH)2-16-ene-5,6-trans-D3 greatly suppresses cell proliferation by causing a block in the G1-G0 cell cycle, linked with an increased expression of numerous cyclin-dependent kinase inhibitors (CDKIs). This process significantly lowers telomerase activity, while having an extremely low calcemic effect [55].

Vitamin D and its analogs have been included in leukemia therapy. It can be used in treating chronic myeloid leukemia (CML), acute lymphoblastic leukemia (ALL), acute myelogenous leukemia (AML), chronic lymphocytic leukemia (CLL), and childhood acute lymphoblastic leukemia (cALL) [49,56,57]. In vivo studies showed decreased levels of this secosteroid circulating in the bloodstream. Supplementation via sufficient doses of calcitriol in cases of CML and ALL and even higher doses in CLL has been linked with the arrest of leukemia development [58]. Moreover, in patients experiencing a relapse, vitamin D level was lowered, compared to those who underwent complete remission [59]. It was also proven that insufficient levels of calcitriol are connected with inferior time-to-treatment and overall survival in CLL patients [60].

#### 3.2.3. Vitamin E

Tocotrienol and tocopherol belong to the vitamin E group. They both comprise four isoforms—alpha, beta, gamma, and delta. Their high levels can be found in some vegetable oils and certain types of seeds, nuts, and grains [61]. Apart from being well-known antioxidants, they display various other properties and act as antitumor, anti-inflammatory, anti-diabetic, neuroprotective, and cardioprotective agents [62]. Tocotrienol’s aforementioned activities surpass tocopherol’s, because of the former’s more effective incorporation into the cell’s lipid layer. Vitamin E activates the pregnane X receptor (PXR), an orphan nuclear receptor. PXR and RXR can combine into a heterodimer, which then binds to particular elements in gene regions and causes a reaction [63].

**Figure 8:**
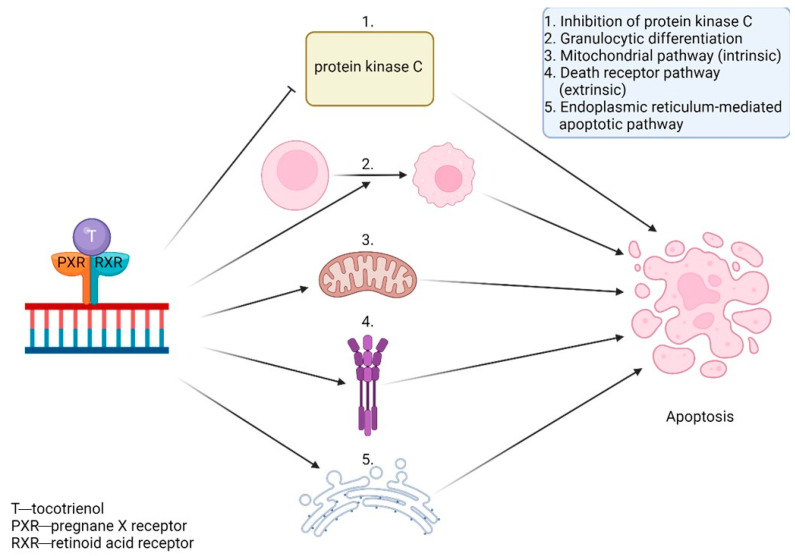
The influence of tocotrienol on DNA, through various pathways, leads to apoptosis.

Studies in vitro, mostly on K562 cells, show tocotrienol’s involvement in reducing telomerase activity. It lowers the expression of *hTERT* and *c-myc* mRNA through protein kinase C inhibition [11]. It also activates apoptosis through the use of various signaling pathways, including the mitochondrial pathway (intrinsic), the death receptor pathway (extrinsic), or other mechanisms like the endoplasmic reticulum-mediated apoptotic pathway. Furthermore, they can activate caspase-3, the release of cytochrome c, and the cleavage of PARP-1 to induce cell death [64]. They also activate granulocytic differentiation [65]. Those apoptotic properties are displayed in Figure 8. Tocotrienol and ferulic acid work synergistically and greatly decrease *hTERT* expression [11].

Vitamin E and its derivatives have been proposed in AML therapy [64]. Patients who were supplemented with vitamin E during their chemotherapy had a great increase in total antioxidant, serum albumin, ceruloplasmin, and immunoglobulin levels with a significant reduction in serum ferritin [66]. It can also be considered in treating chronic myeloid leukemia (CML), where it can act as a leukemic cell differentiation inducer [67]. Clinical trials demonstrate positive results in cancer prevention through supplementing tocopherol and catechol-O-methyltransferase (COMT) [68].

#### 3.2.4. Vitamin C

Ascorbic acid, widely known as vitamin C (VC), is being supplemented by many people every day. It can be found in fruits and vegetables and plays an important role in the human system. This well-known antioxidant helps with neuronal differentiation, reparations of tissue, the formation of collagen, and the activity of the immune system [69,70,71]. Perfluorooctanesulfonate (PFOS) is a global pollutant that can cause toxic effects on various cells, including blood cells, which can result in leukemia. A bioinformatics study on PFOS-associated leukemia showed that VC targets hTERT, amongst other core proteins, which would suggest that it could lower the telomerase activity in vitro and in vivo [72]. A study using CD34^+^, HL-60, and U937 cell lines shows that a high dose of vitamin C stops cell proliferation in a concentration-dependent manner. With higher VC concentration, a greater rate of apoptosis was detected [73].

### 3.3. Fatty Acids and Their Esters

Dietary lipids are composed mainly of fatty acids, which can be saturated or unsaturated. The most well-known polyunsaturated acids are Ω-3 and Ω-6 fatty acids, due to their role in the human body. They are involved in the creation of cell membranes, the regulation of blood pressure, and inflammatory responses. Recent studies also indicate their antitumor properties [74]. They play a vital role in steroid enzyme creation and work as protective agents in cardiovascular disease and dementia [11]. Fatty acids C18–C22 directly affect telomerase activity. Cis-fatty acids surpass trans-fatty acids in effectivity and their inhibitory abilities increase with the number of double bonds, which makes docosahexaenoic acid (DHA) the most effective of them all [75].

Fatty acids inhibit telomerase activity on the transcriptional level. Eicosapentaenoic acid (EPA) shows competitive inhibition toward telomerase substrate primer. This means it can interact with the telomerase primer-binding site. Physiological doses of DHA and EPA (≤50 µM) are shown to inhibit PKC, which causes the down-regulation of *hTERT* and c-Myc mRNA, resulting in lowered telomerase activity [11]. Mono-unsaturated linear-chain fatty acids in the cis configuration with C4A hydrocarbon chains (i.e., oleic acid) also severely inhibit the activity of telomerase. Oleic acid decreases telomerase activity competitively to the telomerase substrate primer [76]. The use of valproic acid (VPA) alone or combined with bortezomib treatment on HL-60 cells resulted in cyclin D1 and *hTERT* inhibition, lowered telomerase activity, arrested proliferation, and triggered apoptosis [77,78].

23-Hydroxybetulinic acid is a substance that can be extracted from *Pulsatilla chinensis.* It is a Chinese medicine herb that can be used in treating malaria and amoebic dysentery. It can also be used as a detoxifying agent. A study on HL-60 shows its apoptotic properties. This acid down-regulates Bcl-2 and lowers telomerase activity [79]. Sodium butyrate also shows anti-cancer abilities. Studies on U937 and HL-60 cells display the inhibition of growth and the induction of apoptosis after the use of this substance. The activity of telomerase was dramatically suppressed, and the expression of *hTERT* was gradually down-regulated [80,81].

Clinical trials and treatment focus on fatty acid properties, which are not connected to telomerase activity. Ω-3 fatty acids effectively target the nuclear factor kappa B (NFκB) pathway in chronic lymphocytic leukemia (CLL), which plays a vital role in the control of apoptosis and progression of hematologic malignancies [82]. It was also used to prevent severe neutropenic enterocolitis in patients with AML, and cardiotoxicity in children with ALL, who are treated with doxorubicin [83,84]. Moreover, it was proven to reduce inflammatory risk in patients undergoing chemotherapy, due to hematological malignancies [85]. VPA can be used in AML treatment, where it acts as a histone deacetylase inhibitor and triggers cytotoxicity toward tumors in AML blasts [86]. VPA and low-dose cytosine arabinoside (Ara-C) indicate positive results in the treatment of elderly patients with AML/RAEB, which means it can be considered an alternative treatment for individuals who are unable to receive standard induction therapy [87].

Trichostatin A is an organic substance with a histone deacetylase activity. In the study performed on the U937 cells, different doses of the substance were added and the cells were incubated for 48 h. The hTERT mRNA expression was inhibited and was the most effective with a concentration of 75 nM, whereas TEP-1 and hTERT mRNA expression remained unchanged [88].

Caffeic acid undecyl ester (CAUE) can be naturally found in *Daphne oleoides* [89]. It displays multiple bioactivities, such as anti-inflammatory, antioxidant, and anti-leukemic properties [90]. A study on B cell precursor leukemia NALM-6 cells shows that CAUE selectively impairs DNA synthesis and lowers the telomerase activity via the suppression of hTERT protein. The higher the concentration of CAUE, the better inhibiting properties it displayed toward hTERT. The noted telomerase activity decrease was to 92% with the use of a 0.1 µM concentration and to 0% with a 1 µM concentration [91].

### 3.4. Substances Used in Traditional Chinese Medicine

There is a great number of substances used in modern medicine that hail from ancient, medieval remedies and traditional Chinese medicine. That latter is full of herbs that are indigenous to their region and were previously used in treating multiple conditions, without fully understanding the mechanisms behind them. Right now, some of them are being used in current leukemia therapies, but there are a lot more perspectives for those compounds, and researching them could provide a lot of new treatment possibilities [92,93]. 

Tanshinone IIA (Tan IIA) is a substance found in *Salvia miltiorrhiza.* It has a long history of usage in traditional Chinese medicine and is now used as a pharmacologically active lipophilic agent. It can be used in the treatment of cancer, obesity, diabetes, and cardiovascular, neurogenerative, and cerebrovascular diseases [94]. A study on K562 and HL-60 cells displayed strongly reduced cell proliferation and lowered activity of telomerase with Tan IIA-induced apoptosis [95]. 

Tan IIA is not the only compound that can be extracted from *Salvia miltiorrhiza*—the other is tanshinone I (Tan I). It is an abietane diterpenoid, which can be used as an anticoronaviral agent. It has therapeutic properties used in treating myeloid leukemia, osteoporosis, left ventricular hypertrophy, hearing loss, hypertension, and atherosclerosis [96]. A study on the monocytic leukemia cells U937, SHI 1, and THP-1 showed Tan I-induced apoptosis through the activation of the caspase-3 pathway and slowed cell growth. This substance lowers the activity of telomerase and down-regulates the expression of hTERT in a dose-dependent manner [97].

Tianshengyuan-1 (TSY-1) is a Chinese herbal medicine that is a liquid mixture of various Chinese herbs. It has been used in treating bone marrow-connected diseases, such as anaplastic anemia [98]. It also affects the activity of telomerase in hematopoietic cells. In a study on mononuclear cells from normal peripheral blood (PBMC), HL-60, and CD34+ cell lines, the mechanism of the TSY-1 effect on telomerase was investigated. In PBMC and CD34+ stem cells, firstly, low telomerase levels increased and in HL-60 initially, high telomerase activity decreased. Those processes happen in a dose-dependent manner. The TSY-1 effects on the activity of telomerase are related to cell senescence. TSY-1 induces the hypomethylation of the core promoter of *TERT* in HL-60 cells and the hypermethylation of the core promoter of *TERT* in PBMC and CD34+ [99]. 

Angelica sinensis polysaccharide (ASP) is a water-soluble substance that can be found in *Angelica sinensis* and is used in traditional Chinese medicine. It has multiple purposes, such as promoting immunity, hematopoietic activity, and liver protection, and can be used as an antitumor, antioxidant, anti-inflammatory, antiviral, and anti-aging agent. Moreover, it can be used as a drug carrier [100]. A study shows that ASP efficiently suppresses the proliferation of AML CD34+CD38− cells in a dose-dependent manner while preserving progenitor and normal hematopoietic stem cells at physiologically attainable doses. Moreover, it exhibits cytotoxic properties on AML K562 cells. ASP represses telomerase activity and up-regulates *p53, p21, p16,* and *Rb* genes, which causes cell senescence [101].

Diterpene triepoxide (triptonide) is a substance that can be acquired from a *Tripterygium wilfordii* Hook F (TwHF). It is used as a Chinese medicinal herb as an immunosuppressive agent and can be used in treating rheumatoid arthritis, psoriasis, and systemic lupus erythematosus [102]. It can act as a reversible non-hormonal contraceptive for male mice and non-human primates [103]. It has anti-cancer activities and is researched as a potential treatment for various neoplasms, like lung, breast, and thyroid cancer [104,105,106]. To test its anti-leukemic properties, a study on AML cell lines was conducted. It was found that triptonide induces absolute senescence in U937 and HL-60 cells, as well as inhibiting growth and colony formation. In a mouse xenograft model, this substance almost completely reduces human leukemia cell tumorigenicity with no evident toxicity. It causes apoptosis by down-regulating the transcription of *TERT* and oncogenic *c-Myc*. At the same time, it promotes the transcription of senescence-promoting genes, such as *p16* and *p21*, as well as pro-apoptotic genes [107].

Wogonin is one of a few active compounds found in *Scutellaria baicalensis* roots. It has been used in Chinese herbal medicine and is being tested in clinical trials now. This substance displays various bioactivities and can be used as an anti-inflammation, anti-cancer, antibacterial, antiviral, neuroprotective, and chondroprotective agent [108,109]. A study on HL-60 showed that wogonin, one of 29 compounds isolated from *Scutellaria baicalensis*, inhibits cell growth mostly via the suppression of telomerase through the inhibition of c-Myc and promotion of Bax/Bcl-2 apoptosis. It inhibits *hTERT*, and human telomerase-associated protein 1 (hTP1), causing a reduction in cell viability in a dose-dependent manner. Moreover, wogonin causes DNA fragmentation, the down-regulation of *c*-myc messenger ribonucleic acid (m-RNA) expression, and caspase-3 activity increase [110]. 

### 3.5. Other Substances

#### 3.5.1. Alkaloids

Camptothecin (CPT) is a cytotoxic alkaloid that can be extracted from *Camptotheca acuminata*. It is known for having broad anti-proliferative and anti-cancer properties due to its ability to inhibit DNA topoisomerase 1. Due to its instability, low solubility, and tumor cells’ developed CPT resistance, a few derivatives were created. There are currently three CPT-inspired pharmaceuticals—topotecan, irinotecan, and belotecan—that are used in the treatment of various types of cancer [111]. They are silicon-containing agents with enhanced lipophilicity, which makes them more bioactive than their carbon analog [112]. In order to analyze its prospective use in leukemia treatment, a study on HL-60 human leukemia cells was conducted. This study shows that CPT decreases telomerase activity in a time-dependent way, whilst inducing apoptosis. It also gradually reduces the expression of Bcl-2, but it has no impact on *TERT* expression [113].

Homoharringtonine (HHT) is a substance derived from the Chinese evergreen *Cephalotaxus harringtonia* and exhibits antitumor activity. W. Xie et al. performed a study on the HL60 cells in order to examine the inhibition of telomerase activity after adding HHT. Different doses (0–500 μg/mL) were added, and the cells were incubated for 48 h. The activity decreased from 1.056 ± 0.107 to 0.067 ± 0.023. The inhibition of telomerase proceeded in a time- and dose-dependent manner [114].

#### 3.5.2. Lactones

Constunolide is a sesquiterpene lactone present in the stem bark of *Magnolia sieboldii* and it exhibits anti-carcinogenic, antiviral, anti-inflammatory, and anti-fungal properties. In the study performed on the NALM-6 cells, 10 μM of constunolide was used, which caused the suppression of telomerase activity to 94%, 89%, 21%, and 2% after 1, 2, 4, and 6 h, respectively. Moreover, hTERT mRNA and hTERT protein expressions decreased. In the case of mRNA, this decrease was to 96, 69, 21, and 20% after 1, 2, 4, and 6 h, respectively, and when it came to the protein, to 97, 69, 21, and 20% after similar times. The effect of caspase inhibitors was also examined during the study. It was concluded that the caspase inhibitors blocked the apoptotic death of the cells (not the caspase 9 inhibitor, however) [115].

Withaferin-A (Wi-A) is a steroidal lactone that can be found in *Withania somnifera* (Ashwagandha). The direct effect of this substance on telomerase is yet to be established, but it has been reported that it has an antitumor and growth-inhibitory effect. Moreover, this substance impairs metastasis and angiogenesis. A study on U937 cells proves that Wi-A induces apoptosis through the activation of caspase-3, Akt signal pathway, and NF-_K_B activity inhibition [116]. Another study on JFCF-6B (TEP—cells with telomerase) and JFCF-1L (ALT—cells without telomerase) showed that Wi-A works on both TEP and ALT. It affects p53 activation, the induction of reactive oxygen species (ROS), the induction of DNA damage, and cytoskeletal dysfunction in both cases. In TEP cells, it also affects telomerase and causes growth arrest, and in ALT cells it results in apoptosis. This study was not conducted on the leukemia cell line, but it describes how Wi-A influences the ALT mechanism, which is observed in leukemia and may be prospective study material [117].

#### 3.5.3. Quinones

β-Lapachone is a substance found in the heartwood of the lapacho tree (*Handroanthus impetiginosus).* It has various biological activities, such as anti-cancer, antiviral, anti-parasitic, anti-inflammatory, anti-obesity, neuroprotective, nephroprotective, antioxidant, and wound-healing effects [118]. A study on the human leukemia cells Hl-60, K562, U937, and THP-1 confirmed direct LAPA-induced cytotoxicity. It activates the subsequent cleavage of poly (ADP-ribose) polymerase and caspase-3. The cell death was connected to lowered telomerase activity and the down-regulation of *hTERT* expression [119]. 

Salvicine is a structurally modified derivative of *Salvia prionitis* that exhibits antitumor activities. In the study, telomerase activity was measured in the HL60 cells after adding 10 μM of salvicine. Telomerase activity was lowered to 77.9, 51.3, and 36.2% after 2, 4, and 6 h, respectively. Meanwhile, the enzyme activity was also measured after 4 h of applying 2.5, 5, 10, and 20 μM of the substance, which caused a decrease to 76.3, 60.1, 40.9, and 32.2% of activity, respectively [120].

#### 3.5.4. Sapogenins

Diosgenin is a natural steroidal sapogenin found in the seeds of fenugreek (*Trigonella foenum-graecum*) and the roots of wild yam (*Dioscorea villosa*) [121]. It has multiple purposes and can be used in treating diabetes, asthma, arthritis, cancer, and cardiovascular disease [122]. A study on chronic myeloid leukemia (CML) cell lines K562 and BaF3-WT displays the strong anti-cancer properties of diosgenin. It generates ROS, which have a cytotoxic effect on CML cells and induce autophagy, which functions as a cytoprotective agent [123]. The direct connection between telomerase and diosgenin was found during research on rat C6 and human T98G glioblastoma cell lines—the study showed the down-regulation of *TERT* expression [121].

#### 3.5.5. Saponins

Ginsenoside Rg1 (Rg1) is a bioactive substance that can be found in the roots of ginseng (*Panax ginseng*) and turned white or red ginseng [124,125]. Rg1 has a hepatoprotective effect, promotes cerebral angiogenesis and lymphatic drainage, improves chronic inflammatory arthritis, and prevents PTSD-like behaviors [126,127,128,129]. A study on CD34+CD38− leukemic stem cells (LSC) showed that Rg1 down-regulates the expression of *hTERT* and up-regulates *p16^INK4a^* (a protein that slows down the cell cycle) expression, which causes senescence of LSC [124]. Another study, using Korean red ginseng (KRG) on U937 cells, showed its anti-cancer properties. KRG has been found to induce apoptosis and down-regulate *hTERT* expression in a concentration-dependent manner [125].

Patensin is a substance derived from *Pulsatilla patens* var. *multifida* and is used in the treatment of amoebic and bacterial diseases, as well as cancers. It causes growth arrest and apoptotic cell death. In the study performed on the HL60 cell line, telomerase activity was measured by the TRAP assay. A dose of 100 μM of patensin was applied and after 3, 6, 12, and 24 h, telomerase activity was down-regulated to 62.98, 44.23, 32.32, and 23.30%, respectively [130].

Platycodin D (PD) is a triterpenoid saponin that can be found in *Platycodon grandiflorus.* It exhibits various bioactive properties and can be used in treating brain ischemia, cardiomegaly, cholestasis, esophagitis, fibrosis, hepatitis C, liver cirrhosis, obesity, osteoporosis, pneumonia, and some kinds of neoplasm [131]. A study on THP-1, K562, and U937 cells was conducted to test PD’s anti-cancer effects. PD has a direct cytotoxic influence on telomerase through a decrease in *hTERT* expression. It reduces the level of c-Myc and Sp1 proteins as well as DNA-binding actions. Moreover, it suppresses activation, which reduces the phosphorylation and nuclear translocation of hTERT. Those findings reveal that PD causes the hTERT post-transcriptional and translational inhibition of telomerase activity [132].

#### 3.5.6. Substances Containing Sulfur

Sulfoquinovosyldiacylglycerol (SQDG) is a substance found in plants and seaweed. The studies indicate its inhibitory effect on human telomerase [133]. It also targets acute lymphoblastic leukemia (ALL) cells by decreasing the catalytic activity of the topoisomerase I enzyme, activating the p53-dependent apoptotic pathway [134]. Moreover, it promotes ataxia telangiectasia-mutated (ATM) and Rad3-related kinase (ATR), which are important parts of the cell cycle checkpoint machinery recruitment at chromatin [135]. Those processes stop cells in the S-phase [134].

Sulforaphane (SFN) is a chemical compound belonging to the isothiocyanate group. It is found naturally in cruciferous vegetables, such as broccoli or Brussels sprouts. In the study performed by H. Shang et al. on the cells from the HL-60 cell line, 8 mM of SFN after 48 h of application down-regulated hTERT gene expression 2.1-fold, which was noted by cDNA microarray analysis [136].

#### 3.5.7. Xanthenes

Crocin is a water-soluble carotenoid pigment that can be found in the stigma of crocuses (*Crocus sativus*) or gardenias. It has four chemical analogs, but due to its pharmacological effects and the amount found in saffron, crocin 1 (alpha-crocin; crocetin digentiobiose ester) has been studied the most. It has multiple properties and participates in the creation of adenosine triphosphate (ATP), signal transduction, and redox homeostasis. Its direct connection with lowered telomerase activity was observed on HepG2 cells, where *hTERT* expression was halted. Its anti-leukemic properties were studied on cell line HL-60 and resulted in inhibited proliferation and induced apoptosis. The crocin treatment of HL-60-xenografted mice reduces tumor weight and size as well as Bcl-2 expression, while increasing Bax expression [137]. Crocin also induces apoptosis and stops cell growth in Jurkat (human T-cell leukemia cell line), which can be an indication of its therapeutic properties in treating T-lineage acute lymphoblastic leukemia (T-ALL) [138].

### 3.6. Substances Produced by Animals

Other interesting discoveries are made by researching substances made by animals—especially those found in marine areas. Even substances produced by human organisms have been proven to have bioactive abilities [139,140,141]. 

Melatonin is a hormone widely known for its control of the sleep–wake cycle [139]. It is synthesized in a human pineal gland but can also be found in a variety of foods, such as eggs, fish, and nuts [142]. Apart from its wide range of bioactive properties, including antioxidant and anti-inflammatory qualities, it also displays antitumor effects. Studies on cell lines RS4-11 (MLL-AF4+ B-ALL), Nalm-6 (non-MLL-r B-ALL), and MOLM-13 (MLL-AF9+ AML) show the melatonin-induced suppression of mixed lineage leukemia (MLL). This substance inhibits *hTERT* expression by invalidating the binding activity of RBFOX3 to the *hTERT* promoter, which ceases cell proliferation. Moreover, it blocks NF-κB nuclear translocation and inhibits NF-B binding to the COX-2 promoter, causing lower COX-2 production. Clinical data showed that melatonin displays an anti-leukemic effect in primary MLL-r leukemia blasts ex vivo. In an MLL-r leukemia xenograft mouse model, animals treated with melatonin had a greater decrease in leukemic burden than the control group [139].

Interferon alpha (IFN-α) is a protein made in the human body against viral infections, mainly by plasmacytoid dendritic cells (pDCs). It has been used in the treatment of myeloproliferative neoplasms (MPNs) and has been shown to induce hematological, histopathological, and molecular responses [140]. Although IFN-α has various impacts on stem cells and immunology, the mechanism of its anti-leukemic activity is still unknown. Numerous clinical trials have shown that chronic myeloid leukemia treatment with IFN-α had a higher number of remissive patients than traditional chemotherapy did. From the 1980s until 2001, it has been the first line of treatment for CML—right now, the combination of IFN-α [EB1] and other drugs is being tested [143]. It was also tested as maintenance therapy on patients with favorable-risk AML and showed positive results [144]. IFN-α has also been shown to amplify graft-versus-leukemia (GVL) response in the case of allogeneic hematopoietic cell transplantation (HCT) for high-risk AML, which prevents leukemia relapse [145].

Manisa propolis is a wax-like substance collected by honeybees. It is believed to have anti-microbial, anti-inflammatory, antioxidant, and antitumoral properties. O. Cogulu et al. examined the impact of propolis on four childhood leukemia cases—three of which were acute lymphoid leukemia and one was chronic myeloid leukemia. At 24 h after applying 60 ng/mL of propolis, the expression level of *hTER* decreased [146]. In another study performed on the T-cell ALL cells (CCFR-CEM), 0.03 μg/mL was added to cells, which resulted in a decrease in the hTERT ratio by 60 and 93% at 24 and 72 h, respectively [147].

#### 3.6.1. Substances Produced by Organisms from Marine Ecosystems

Pectenotoxin-2 (PTX-2) is a natural toxin that can be isolated from marine sponges (*Dinophysis species*). They can affect actin cytoskeletons in vivo and in vitro. They display cytotoxicity toward various cancer cells, such as lung, colon, and breast cancer cells. A study on U937, HL-60, and THP-1 cells showed that PTX-2 decreases the viability of cells and inhibits telomerase activity via the suppression of *hTERT* expression. It reduces *Sp1* and *c-Myc* gene expressions and lowers the binding activity of their proteins to the *hTERT* regulatory regions. It also reduces the phosphorylation and nuclear translocation of hTERT via the attenuation of the phosphorylation of Akt. This concludes that PTX-2 suppresses telomerase activity through the post-transcriptional and translational inhibition of hTERT [141].

Dideoxypetrosynol A is a polyacetylene present in the marine sponge *Petrosia* sp. It has shown cytotoxicity toward several human cancer cell lines. In the study performed by K. Mandel et al. on the U937 cell line, different concentrations of dideoxypetrosynol A were added and the cells were incubated for 48 h. The down-regulation of hTERT mRNA was observed at 0.6 μg/mL; however, there was no effect on hTERT, TEP-1, and c-myc mRNA [148].

(Z)-stellettic acid C is an acetylenic acid present in the *Stelletta* sp. sponges. It has shown selective cytotoxicity toward human cancer cell lines. In the study on U937 cells, telomerase activity was inhibited in a concentration-dependent manner and was the most effective at 30 μg/mL; however, there was no change in TEP-1 and hTERT mRNA expressions. Similarly, (Z)-stellettic acid C had no effect on the crucial transcription factors for the regulation of transcription of hTERT—Sp-1 and c-Myc [149].

*Gymnodinium* sp.* A3 (GA3)* is a marine microalga that was first isolated from the Seto Inland Sea. When this dinoflagellate grows in saltwater, it produces extracellular acidic GA3 polysaccharide, which is a D-galactan sulfate associated with L(+)-lactic acid. This substance demonstrated significant cytotoxicity to various human leukemic cell lines [150]. A study on K562 cells displays GA3 polysaccharides’ ability to lower telomerase activity [151]. Moreover, it inhibits topoisomerase-I and topoisomerase-II, resulting in apoptosis [150]. 

#### 3.6.2. Substances Produced by Bacteria

Telomestatin is a new inhibitor of telomerase that can be extracted from *Streptomyces anulatus.* It has great inhibitory properties and affects telomerase without influencing polymerases and reverse transcriptases. This substance also promotes the shortening of telomeres [152]. A study on U937 cells showed the activation of the telomestatin-induced caspase-3 pathway and apoptosis due to telomerase inhibition. The growth of cells was also slowed in U937 cells in the xenograft mouse model, thanks to decreased telomerase levels. Moreover, telomestatin suppresses the activity of the telomerase in BCR-ABL-positive leukemic cell lines in a reproducible manner [153]. 

### 3.7. Substances Produced by Fungi

Cordycepin (3′-deoxyadenosine) is an adenosine nucleoside derivative that can be found in the fungus *Cordyceps militaris*. It has broad therapeutic potential and can be used as an antioxidant, anti-cancer, anti-diabetic, antiviral, inflammatory, hepatoprotective, anti-inflammatory, anti-aging, and anti-arthritic drug [154]. A study on human leukemia cells THP-1 and H937 showed that cordycepin-induced apoptosis influences telomerase, causing its deactivation and the down-regulation of both hTERT and its transcription factors—c-Myc and Sp1. Moreover, cordycepin inhibits the phosphoinositide-3-kinase (PI3K)/protein kinase B (Akt) pathway, which lowers the phosphorylation and translocation of hTERT [155].

*Agaricus blazei Murill* (AbM) is an edible mushroom found in Brazil. It exhibits various effects, such as antitumor, anti-inflammatory, anti-allergic, anti-HIV, hepatoprotective, and antioxidant effects [156,157,158]. An RNA–protein complex, FA-2-b-ß fraction, can be isolated from AbM. The anti-leukemic and pro-apoptotic effects of this substance were tested on HL-60 cells. FA-2-b-ß fraction causes a decrease in telomerase activity and induces *caspase-3* gene expression, which may lead to apoptosis [159]. 

**Table 1:** Substances used in this review.

Substance	Natural Occurrence	Cell Line	Dose Applied	Durance	IC_50_ or ED_50_	
Alkaloids						
Camptothecin	*Camptotheca acuminata*	HL-60	1 mg/L	2, 4, 6 h	not specified	[113]
Homoharringtonine	Chinese evergreen *Cephalotaxus harringtonia*	HL-60	5, 10, 50, 100, 500 μg/L	3, 6, 12, 24, 48 h	not specified	[114]
Bacteria						
Telomestatin	*Streptomyces anulatus*	U937	1, 2, 5, 10 μM	48 h, 0–60 days	not specified	[153]
Fatty acids and their esters						
23-Hydroxybetulinic acid	*Pulsatilla chinensis*	HL-60	1–1000 μM	3, 6, 12, 24 h	not specified	[79]
		HL-60, HL-60A	0.5, 1, 2, 4, 8 mM	24, 48, 72 h	2.83, 3.90 mM	[77]
Valproic acid	*Valeriana officinalis*	18 + 114 patients of AML	400 mg/day + from day 3, 60–150 mg/L	2 × 21 days, 3 × 21 days	not specified	[86]
		31 cases of elderly AML/RAEB	5 mg/kg daily	2–23 months	not specified	[87]
Ω-3 fatty acids (DHA, EPA)	Seafood, especially fatty fish, nuts, seeds, plant oils	16 cases of Rai Stage 0–1 CLL	1. month: 3 × 1250 mg/day 2. month: 6 × 1250 mg/day 3.–12. month: 9 × 1250 mg/day	12 months	not specified	[82]
		14 cases of AML	100 mL/day	2 years	not specified	[84]
		60 cases of cALL (30 study/30 control)	1000 mg/day	6 months	not specified	[83]
Caffeic acid undecyl ester	*Daphne oleoides*	NALM-6	0.1, 0.3, 0.6, 1, 3 μM	4, 18 h	not specified	[91]
		NALM-6	0.1, 0.3, 0.6, 1 μM	6, 12, 24, 72 h	0.33, 0.16 μM	[90]
Trichostatin A	Culture broth of *Streptomyces platensis*	U-937	15, 30, 45, 60, 75 nM	48 h	not specified	[88]
Fungi						
Cordycepin	*Cordyceps militaris*	THP-1, H937	10, 20, 30 μM	24 h	not specified	[155]
FA-2-b-ß fraction (an RNA-protein complex)	*Agaricus blazei Murill*	HL-60	5, 10, 20, 40, 80 μg/mL	24, 48, 72, 96 h	211, 187.35, 89.61, 42.72 µg/mL	[159]
Lactones						
Constunolide	Stem bark of *Magnolia sieboldii*	NALM-6	2, 6, 8, 10 μM	1, 2, 4, 6 h	3.31 ± 0.32 mM	[115]
Withaferin-A	*Withania somnifera*	U937	0.25, 0.5, 1, 2 μM	12, 24 h	not specified	[116]
Polyphenols						
Curcumin	*Curcuma longa*	HL-60	1, 10, 50 μM	24 h	9.8 μM	[23]
		HL-60	10, 15, 20, 40 μM	24 and 48 h	not specified	[24]
		K-562	1, 10, 50 μM	6, 16, 24, 48 h	14 μM	[25]
Epigallocatechin-3-gallate (EGCG)	*Camellia sinensis*	HL-60	50 μM	3, 6, 9, 12 days	not specified	[26]
		Jurkat	30, 50, 70, 85, 100 μM	24, 48, 72 h	not specified	[27]
Indole-3-carbinol	*Brassica oleracea*	NALM-6	20, 30, 40, 50, 60 μM	24, 48 h	not specified	[29]
		K-562	100, 200, 400 μM	24, 48 h	not specified	[30]
Butein	Stem bark of *Anacardium occidentale*, the heartwood of *Dalbergia odonifea*, *Rhus verniciflua*, and *Caragena jubata*	U937, THP-1, HL60, K562	5, 10, 15, 20, 30 μM	24 h	not specified	[31]
Genistein	*Fabaceae*	HL-60	50 μM	24, 48, 72 h	50 μM	[17]
Apigenin	*Lamiaceae, Allium cepa, Petroselinum crispum*, wine, tea, beer	HL-60, U937, THP-1	25, 50, 75, 100 μM	24 h	not specified	[33]
Silymarin	*Silybum marianum*	K562	10, 25, 50, 75, 100 μg/mL	24, 48, 72 h	not specified	[35]
Baicalin	*Scutellaria baicalensis*	HL-60	HL-60 5, 10, 20, 40, 80 μg/mL	12, 24, 48 h	21.8 μg/mL	[36]
Quinones						
β-Lapachone	*Handroanthus impetiginosus*	U937, K562, HL60, THP-1	1, 2, 3, 4 μM	24 h	not specified	[119]
Salvicine	*Salvia prionitis* (modified)	HL-60	2.5, 5, 10, 20 μM	2, 4, 6 h	not specified	[120]
Sapogenin						
Diosgenin	*Trigonella foenum-graecum*	K562	5, 10, 15, 20, 30 μM	48 h	not specified	[123]
Saponins						
Ginsenoside Rg1	*Panax ginseng*	CD34+CD38-LSCs	20, 40, 80 μM	24, 48, 72 h	not specified	[124]
		U937	0.2, 0.4, 0.6, 0.8, 1, 1.2, 1.4, 1.6, 0.8, 2 mg/mL	24, 48, 72 h	not specified	[125]
Patensin	*Pulsatilla patens var. multifida*	HL-60	0.001, 0.01, 0.1, 1, 10 mmol/L	3, 6, 12, 24 h	not specified	[130]
Platycodin D	*Platycodon grandiflorus*	U937, THP-1, K562	5, 10, 15, 20 μM	48 h	not specified	[132]
Substances produced by animals						
D-galactan sulfate	*Gymnodinium* sp.* A3*	K562	0.003, 0.01, 0.03, 0.1, 0.3, 1 μg/mL	15, 45 min	not specified	[150]
Dideoxypetrosynol A	Marine sponge *Petrosia* sp.	U-937	0.2, 0.4, 0.6, 0.8, 1 μg/mL	48 h	not specified	[148]
Interferon alpha	Synthesized in the human organism by plasmacytoid dendritic cells	42 study/42 control of AML	3 × week 3 mln IU	12–18 months	not specified	[144]
		36	−1. before HCT, 14., 28., 42. (+/−7) days 45, 90, 180 μg	24 months	not specified	[145]
Manisa propolis	Substance collected by honeybees	4 childhood leukemia cases (3 ALL, 1 CML)	15, 30, 60 ng/mL	24, 48, 72 h	not specified	[146]
		CCFR-CEM	0.03 μg/mL	24, 48, 72, 96 h	0.03 μg/mL	[147]
Melatonin	Synthesized in a human pineal gland, eggs, fish, nuts	RS4-11 (MLL-AF4+ B-ALL), MOLM-13 (MLL-AF9+ AML), Nalm-6 (non MLL-r B-ALL)	1, 2, 3 mM	24, 48, 72 h	1.523, 0.957, 1.869, 0.728, 0.748, 1.877 mM	[139]
Pectenotoxin-2	*Dinophysis* species	U937, THP-1, HL-60	2, 4, 6, 8, 10 ng/mL	72 h	not specified	[141]
(Z)-stellettic acid C	*Stelletta* sp. sponges	U-937	5, 10, 20, 30, 40 μg/mL	48 h	not specified	[149]
Substances containing sulfur						
SQDG	*Azadirachta indica*	MOLT-4	5, 10, 15, 20, 25 μM	2, 4, 8, 12, 16, 20, 24, 48, 72 h	15.32 ± 0.58, 22.52 ± 0.64, 19.63 ± 0.23, 75.67 ± 6.4 μM	[134]
Sulforaphane	Cruciferous vegetables	HL-60	6, 7, 8, 9, 10 μM	24, 48 h	not specified	[136]
Traditional Chinese medicine						
*Angelica sinensis* polysaccharide	*Angelica sinensis*	K562, CD34+CD38−	20, 40, 80 μg/mL	48 h	not specified	[101]
Tanshinone I	*Salvia miltiorrhiza*	U937, THP-1, SHI 1	10, 20, 30, 40, 50 μM	24, 48, 72 h	not specified	[97]
Tanshinone IIA	*Salvia miltiorrhiza*	HL-60, K562	0.5 μg/mL	5, 6 days	not specified	[95]
Tianshengyuan-1	A mixture of various Chinese herbs	HL-60, PBMCs, CD34+ HSCs	31.2, 62.5 μg/mL	24 h	not specified	[99]
Triptonide	*Tripterygium wilfordii* Hook F	U937, HL-60	0–10, 15, 20 nM 2, 4 mg/kg	72 h, 3, 6, 9, 14, 21 days	7.5, 12 nM	[107]
Wogonin	*Scutellaria baicalensis*	HL-60	0.5, 1, 2, 3 mg/mL 10, 25, 50, 75, 100 μM	24 h	not specified	[110]
Vitamins						
Ascorbic acid	Citrus fruits, green and leafy vegetables	CD34+, HL-60, U937	8, 20 mM	24 h	not specified	[73]
Retinoic acid	Yellow, green, red, and leafy vegetables, yellow fruits	HL-60	1 μM	12 days	not specified	[40]
		242 cases of APL	60 mg/kg/day	2 years	not specified	[160]
		124 cases of cAPL	25 mg/m^2^/day	5 years	not specified	[161]
		Eμ-TCL1 mice	1 μM	24, 48 h	not specified	[47]
9cUAB30	Novel retinoid	HL-60d	5 μM	12 days	not specified	[162]
Growth factor binding protein 7	Human insulin recombinant	HL60, KG1a, THP1, HEK293T	100 μg/mL, 300 μg/mL, 10 mg/kg, 12 mg/kg	48, 72, 120 h, 7, 14 days, 16 weeks	not specified	[42]
Vitamin D	Produced in the human body form 7-dehydrocholesterol; fish oil, egg yolk	HL-60	10^−8^ M	3 days	not specified	[163]
		U937	10^−9^–10^−7^ M	48, 72 h	not specified	[164]
		26 cases of AML	1 μg/day	2 × 5 weeks	not specified	[165]
		17 cases of elderly AML	100,000 IU/week	6 months	not specified	[166]
EB 1089	Vitamin D3 analog	HL-60, U937	5 × 10^−10^ M	96 h	not specified	[54]
1,25(OH)2-16-ene-5,6-trans-D3	Vitamin D3 analog	HL-60	10^−7^ M	4 days	1.9 × 10^−12^ M	[55]
Vitamin E	Vegetable oil, seeds, nuts, grains	U937, KG-1	10–50 μM	24 h	29.43, 25.23 µM	[64]
		K562	100 μM	48 h	not specified	[167]
		25 cases of AML	400 IU/day	30 days	not specified	[66]
		2396 study/2235 control	600 IU/day	10 years	not specified	[68]
Xanthene						
Crocin	*Crocus sativus*	Jurkat	0.625, 1.25, 2.5, 5, 10 mg/mL	12, 24, 26, 48 h	not specified	[138]

**Table 2:** Summary of substances used in this review (all substances have been downloaded from https://pubchem.ncbi.nlm.nih.gov/ (accessed on 12 December 2023)).

Group	Substance	Structure	Properties
Alkaloids	Camptothecin	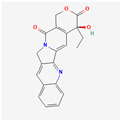	anti-proliferative, anti-cancer DNA topoisomerase 1 inhibition apoptosis induction telomerase activity decrease Bcl-2 expression reduction
	Homoharringtonine	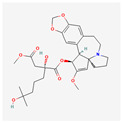	antineoplastic, anticoronaviral protein synthesis inhibition apoptosis induction telomerase activity inhibition
Bacteria	Telomestatin	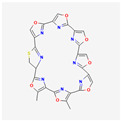	anti-leukemic telomerase inhibition caspase-3 pathway activation apoptosis induction
Fatty acids and their esters	23-Hydroxybetulinic acid	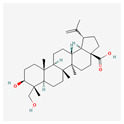	detoxification, anti-malaria, anti-amoebic Bcl-2 down-regulation telomerase activity decrease apoptosis induction
	Caffeic acid undecyl ester	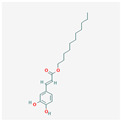	anti-inflammatory, antioxidant, anti-leukemic DNA synthesis impairment hTERT protein suppression telomerase activity decrease
	Docosahexaenoic acid	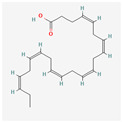	antitumor, cell membrane and steroid enzyme creation inflammatory responses and blood pressure regulation telomerase substrate primer inhibition PKC inhibition hTERT and c-Myc mRNA down-regulation telomerase activity decrease apoptosis and progression of hematologic malignancies control neutropenic enterocolitis and cardiotoxicity prevention
	Eicosapentaenoic acid	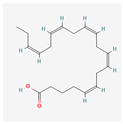	
	Oleic acid	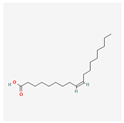	antioxidant telomerase substrate primer inhibition telomerase activity decrease
	Sodium butyrate	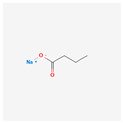	growth inhibition apoptosis induction telomerase activity suppression expression of hTERT down-regulation
	Trichostatin A	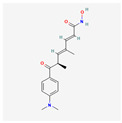	histone deacetylation hTERT mRNA expression inhibition
	Valproic acid	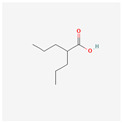	cyclin D1 and hTERT inhibition telomerase activity decrease proliferation arrest apoptosis induction histone deacetylase inhibition
Flavones	Apigenin	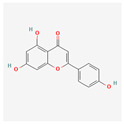	antioxidant, anti-proliferative, antimetastatic, anti-inflammatory caspase pathway activation hTERT expression suppression apoptosis induction telomerase activity decrease intracellular level of ROS increase
	Baicalin	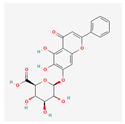	hepatoprotection, antibacterial, anti-inflammatory c-Myc mRNA and protein levels down-regulation hTERT mRNA expression decrease
	Genistein	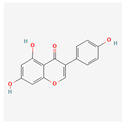	antioxidant, antineoplastic, antitumor, antihelmintic tyrosine kinase inhibitor hTERT gene down-regulation
Flavonolignans	Silymarin	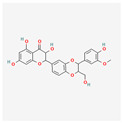	immunomodulatory, anti-cancer, anti-inflammatory, hepatoprotection telomerase activity decrease cell growth suppression apoptosis induction
Fungi	Cordycepin	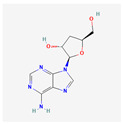	antioxidant, anti-cancer, antiviral, anti-inflammatory, hepatoprotection, anti-diabetic, anti-aging apoptosis induction hTERT, c-Myc and Sp1 deactivation and down-regulation PI3K/Akt pathway inhibition hTERT phosphorylation and translocation decrease
	FA-2-b-ß fraction (an RNA-protein complex)		antitumor, anti-inflammatory, anti-allergic, anti-HIV, hepatoprotection, antioxidant, anti-leukemic, pro-apoptotic telomerase activity decrease caspase-3 gene expression induction
Lactones	Constunolide	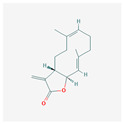	anti-carcinogenic, antiviral, anti-inflammatory, anti-fungal telomerase activity suppression hTERT mRNA and hTERT protein expression decrease
	Withaferin-A	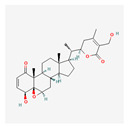	antitumor, metastasis, and angiogenesis impairment caspase-3 and p53 activation growth, Akt signal pathway, and NF-KB activity inhibition apoptosis, ROS, cytoskeletal dysfunction, and DNA damage induction
Polyphenols	Butein	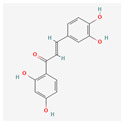	antitumor, pro-apoptotic telomerase activity inhibition hTERT gene transcription suppression Akt inactivation hTERT phosphorylation
	Curcumin	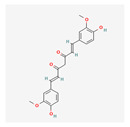	anti-inflammatory, anti-carcinogenic, anti-mutagenic, anti-proliferative apoptosis induction cytochrome c release from mitochondria Bcl-2 expression decrease Bax level increase caspase 3 and caspase 8 activation hTERT post-translational translocation and telomerase inhibition
	Epigallocatechin-3-gallate	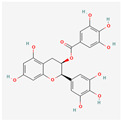	apoptosis induction hTERT gene down-regulation
	Indole-3-carbinol	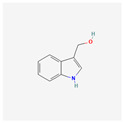	telomerase activity inhibition apoptosis and wild-type p53 induction hTERT gene and c-Myc down-regulation
Quinones	β-Lapachone	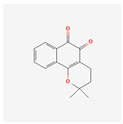	anti-cancer, antiviral, anti-parasitic, anti-inflammatory, anti-obesity, neuroprotective, nephroprotective, antioxidant, wound healing subsequent cleavage of poly (ADP-ribose) polymerase and caspase-3 activation telomerase activity decrease hTERT expression down-regulation cell death induction
	Salvicine	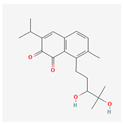	antitumor telomerase activity decrease
Sapogenins	Diosgenin	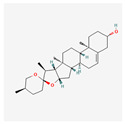	anti-cancer, anti-diabetic, anti-asthmatic, anti-arthritic pro-cardiovascular ROS generation autophagy induction TERT expression down-regulation
Saponins	Ginsenoside Rg1	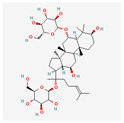	hepatoprotection, cerebral angiogenesis and lymphatic drainage promotion, PTSD-like behavior prevention hTERT expression down-regulation p16INK4a expression up-regulation apoptosis induction
	Patensin		anti-amoebic, antibacterial, anti-cancer growth arrest and apoptosis induction telomerase activity down-regulation
	Platycodin D	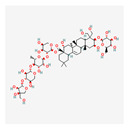	anti-cardiomegaly, anti-cholestatic, anti-fibrotic, anti-obesity, anti-osteoporotic, antineoplastic hTERT expression decrease DNA binding, c-Myc, and Sp1 protein reduction hTERT post-transcriptional and translational inhibition telomerase activity decrease
Substances produced by animals	D-galactan sulfate		telomerase activity decrease topoisomerase-I and topoisomerase-II inhibition apoptosis induction
	Dideoxypetrosynol A	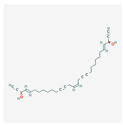	hTERT mRNA down-regulation
	Interferon alpha	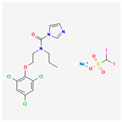	antineoplastic, anti-leukemic, antiviral, anti-proliferative, immunomodulatory
	Manisa propolis		anti-microbial, anti-inflammatory, antioxidant, antitumor hTERT expression decrease
	Melatonin	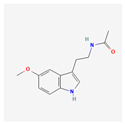	sleep-wake cycle control, antioxidant, anti-inflammatory, antitumor, anti-leukemic binding activity of RBFOX3 to the hTERT promoter invalidation hTERT expression inhibition cell proliferation arrest NF-κB nuclear translocation and NF-B binding to the COX-2 promoter inhibition
	Pectenotoxin-2	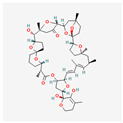	actin cytoskeleton regulation, anti-cancer Sp1 and c-Myc gene expression reduction binding activity of their proteins to the hTERT regulatory regions decrease Akt phosphorylation hTERT post-transcriptional and translational inhibition telomerase activity and cell viability decreases
	(Z)-stellettic acid C	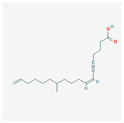	telomerase activity inhibition
Substances containing sulfur	SQDG	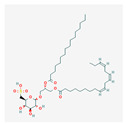	topoisomerase I activity decrease p53-dependent apoptotic pathway activation cell in S-phase arrest telomerase inhibition
	Sulforaphane	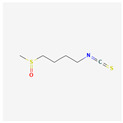	hTERT gene expression down-regulation
Substances used in traditional Chinese medicine	*Angelica sinensis* polysaccharide		pro-immunity, pro-hematopoietic, hepatoprotection, antitumor, antioxidant, anti-inflammatory, antiviral, anti-aging proliferation suppression telomerase activity repression p53, p21, p16, and Rb genes up-regulation
	Tanshinone I	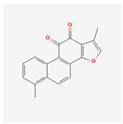	anticoronaviral, anti-leukemic, anti-osteoporotic, antihypertensive, anti-atherosclerotic apoptosis induction caspase-3 pathway activation telomerase activity and cell growth decrease the hTERT expression down-regulation
	Tanshinone IIA	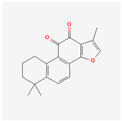	anti-cancerous, anti-obesity, anti-diabetic, anti-neurogenerative, pro-cardiovascular cell proliferation reduction telomerase activity decrease apoptosis induction
	Tianshengyuan-1		anti-hematological core promoter of TERT hypomethylation telomerase activity decrease
	Triptonide	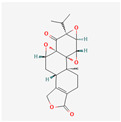	immunosuppression, contraception, anti-cancer, anti-leukemic growth and colony formation inhibition tumorigenicity reduction transcription of TERT and c-Myc down-regulation transcription of senescence-promoting genes, (p16 and p21) and pro-apoptotic genes promotion absolute senescence and apoptosis induction
	Wogonin	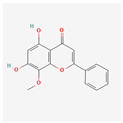	anti-inflammation, anti-cancer, antibacterial, antiviral, neuroprotection, chondroprotection cell growth, c-Myc and hTERT inhibitions telomerase suppression DNA fragmentation induction caspase-3 activity increase Bax/Bcl-2 apoptosis promotion
Vitamins	Ascorbic acid	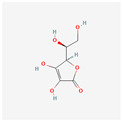	antioxidant, neuronal differentiation, immunomodulatory, tissue repairment, collagen formation apoptosis induction hTERT control
	Calcitriol	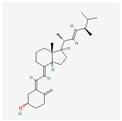	anti-cancer, growth inhibition, apoptosis activation, anti-proliferation, cell differentiation, cancer cells sensitization (their easier cellular intake) telomerase activity, time-to-treatment, overall survival, pro-inflammatory cytokine, and calcemic complications decreases mineralization, insulin secretion, cell maturation, and blood pressure regulation VDR-RXR binds to VDRE in DNA, causing hTERT suppression tumor necrosis factor-α protein (TNF-α) elevation endogenous nitric oxide generation G1-G0 cell cycle blockage cyclin-dependent kinase inhibitors increase leukemia development arrest
	Retinoic acid	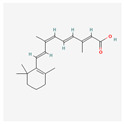	embryonic development and growth, formation of organs, immune function, vision, and reproduction control telomerase inhibition, anti-proliferation, anti-leukemic, apoptosis activation, differentiation, and cell growth arrest RAR and RXR bind to RAREs, causing abrupt hTERT gene transcription reduction differentiation through the hTERT gene down-regulation leukemia stem/progenitor cells eradication susceptibility to ATRA increases through GFI1 expression decrease
	Tocotrienol	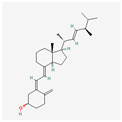	antioxidant, antitumor, anti-inflammatory, anti-diabetic, neuroprotective, cardioprotective, cell’s lipid layer, cell differentiation activation, cancer prevention PXR and RXR bind to DNA, causing protein kinase C inhibition and hTERT and c-myc mRNA decreases telomerase activity reduction apoptosis induction by various signaling pathways (intrinsic, extrinsic, etc.) Bcl-2 protein up-regulation caspase-3 activation, cytochrome c release, and PARP-1 cleavage
Xanthenes	Crocin	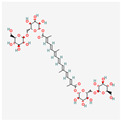	ATP creation, signal transduction, redox homeostasis, anti-leukemic telomerase activity decrease hTERT expression termination proliferation and cell growth inhibition apoptosis induction Bcl-2 expression, tumor’s weight, and size reduction Bax expression increase

## 4. Conclusions

The inhibitory activity of natural substances toward telomerase is gaining more attention from researchers. Telomerase, a key enzyme responsible for maintaining telomere length and promoting cellular immortality, is often up-regulated in leukemia cells, contributing to their uncontrolled proliferation and survival. Through the utilization of natural substances such as plant-derived compounds, researchers have identified potential inhibitors of telomerase activity, offering a targeted approach to treating leukemia. Furthermore, the use of natural substances may offer additional benefits such as minimal side effects and improved patient tolerance compared to conventional chemotherapy.

Experimental studies carried out in vitro and in vivo indicate their significant potential to reduce the development of leukemias. Moreover, the results presented in the paper indicate that natural substances can exert their anti-leukemic effects in various ways. These natural inhibitors demonstrate the ability to disrupt telomerase function, leading to telomere shortening, cellular senescence, and ultimately, the inhibition of leukemia cell growth. They can also induce apoptosis and the differentiation of the cells. 

In our opinion, vitamins are the most promising substances in targeting telomerase in leukemia cells, as they have been studied both in vitro and in clinical studies with good results. Not only that, but also, vitamin D is effective in different types of leukemias, which makes it quite versatile.

However, further research is warranted to fully elucidate the efficacy, safety, and clinical potential of natural telomerase inhibitors in the management of leukemia. There are too few research papers to decide whether or not natural compounds are, indeed, sufficient in reducing the development of leukemias and in treating them. Additionally, it should be mentioned that apart from the benefits, there may also be several limitations associated with the use of natural substances, such as the following:

- Their ambiguous effect on chemoprevention;
- The lack of data indicating the optimal and toxic doses;
- The lack of data regarding their potential side effects;
- The lack of data evaluating their pharmacodynamic properties.

Nonetheless, the inhibition of telomerase via natural substances presents a promising strategy that could complement existing therapeutic approaches and enhance outcomes for leukemia patients in the future.
